# Susceptibility of the Iranian population to severe acute respiratory syndrome coronavirus 2 infection based on variants of angiotensin I converting enzyme 2

**DOI:** 10.2217/fvl-2020-0160

**Published:** 2020-08-17

**Authors:** Alireza Mohebbi, Fatemeh Sana Askari, Mohsen Ebrahimi, Mana Zakeri, Mohammad Yasaghi, Hanieh Bagheri, Naeme Javid

**Affiliations:** 1 ^1^Department of Microbiology, School of Medicine, Golestan University of Medical Sciences, Gorgan, Iran; 2 ^2^Student Research Committee, School of Medicine, Golestan University of Medical Sciences, Gorgan, Iran; 3 ^3^Neonatal & Children’s Health Research Center, Golestan University of Medical Sciences, Gorgan, Iran; 4 ^4^Department of Biology, Islamic Azad University of Tehran, Medical Branch, Tehran, Iran

**Keywords:** human angiotensin-converting enzyme 2, molecular docking, natural resistance variant, SARS-CoV-2, SARS-CoV-2 spike glycoprotein

## Abstract

**Background:** Variations in the viral receptor human angiotensin-converting enzyme 2 (ACE2) may specify the susceptibility of a certain population to severe acute respiratory syndrome coronavirus 2. **Objective:** Evaluation of the affinity of severe acute respiratory syndrome coronavirus 2 spike glycoprotein to the Iranian genetic variants of ACE2. **Materials & methods:** Single nucleotide polymorphisms of ACE2 among the Iranian population were collected from the Iranome database. Missense mutations in the N-terminal peptidase domain were selected for *in silico* analysis. **Results:** 17 missense single nucleotide polymorphisms were found at ACE2. Viral glycoprotein had the lowest affinity to ACE2 mutant V485L. **Discussion:** The V485L variant of ACE2 could be a natural resistance mutation among the Iranian population. In addition, variant S331F can increase slightly the susceptibility to infection with the virus.

The severe acute respiratory syndrome coronavirus 2 (SARS-CoV-2) is a newly emerged coronavirus, first identified in Wuhan, China, and has spread rapidly across the world with over 3 million cases and >200,000 deaths (www.worldometers.info/coronavirus/) [1]. According to research, SARS-CoV-2 is closely related to SARS-CoV; ∼76% of nucleotide sequences between SARS-CoV and SARS-CoV-2 are similar. A total of 94.4% similarity was also found between SARS-CoV-2 and SARS-CoV by protein sequence analysis of seven preserved viral nonstructural protein domains [2]. It is a novel enveloped, nonsegmented virus with a positive sense single-stranded RNA genome (26–32 kb). The SARS-CoV2 proteins contain nucleocapsid (N), envelope (E), membrane (M) and spike (S) [3,4]. S protein is made up of S1 and S2 domains, which play an important role in the virus’ entry into the host cell and is responsible for binding to its host cell receptor, angiotensin-converting enzyme 2 (ACE2) and fusion [5,6].

The ACE2 gene is located on chromosome Xp22 and its product is expressed in large amounts in human lung and small intestine epithelia [7,8]. ACE2 is a Type I transmembrane protein which acts as a monocarboxypeptidase by having a single catalytic active ectodomain, hydrolyzing different peptides [9]. ACE2’s C-terminal domain regulates the transportation of amino acids to the cell surface, making ACE2 an effective receptor for the virus’ attachment and entry into the cell [7,10–12]. By binding to ACE2, S protein cleaves into two separate subunits S1 and S2. The S1 subunit contains a receptor binding domain that binds directly to the ACE2 peptidase domain (PD) and the S2 subunit is used for membrane fusion [13,14].

ACE2 contains a structural transmembrane domain that connects the protein to the plasma membrane. In addition, SARS-CoV-2 S protein interacts with the extracellular domain of ACE2 as a receptor [15]. Interaction between the ACE2 receptor and the S protein is a critical step, as it is the path of absorption of the virus. The presence of variants in ACE2 may therefore reduce or increase its tendency to bind to S protein and may also affect the sensitivity of the host to infection [16,17]. The pattern of expression of human ACE2 and its variations in different populations may be diverse and clear information from all populations is not available. We have therefore decided to provide novel knowledge on the impact of different ACE2 polymorphisms and the affinity of SARS-CoV2 spike glycoprotein. Such knowledge offers a deeper understanding of the virus attachment sensitivity to a certain polymorphism in ACE2.

## Materials & methods

### Construction of SARS-CoV-2 glycoprotein

Prediction of 3D protein structures from amino acid sequence SARS-CoV-2 glycoprotein (accession numbers: YP_009724390.1) was performed by using I-TASSER server (http://zhang.bioinformatics.ku.edu/I-TASSER) [18]. In comparison with homology-based tools, I-TASSER server generates full-length 3D protein structure that enables one to make amino acid changes. The best model with highest confidence score was used for the study.

### Retrieve of human ACE2 & reconstruction of the mutated proteins

The sequences and validated crystallographic structure of ACE2 was obtained from UniProt (www.uniprot.org) and protein databank (PDB; www.rcsb.org/pdb) with ID:1r42, respectively [19,20]. The crystallographic structure of ACE2 cleared for the presence of water atoms and other unnecessary extra chains by using UCSF Chimera 1.10.2 [21].

Iranome (www.iranome.com) [22], a database containing whole exome sequencing of 800 individuals from eight major Iranian ethnic groups, was used to extract different ACE2 variants and their respective frequencies among the Iranian population. Missense substitutions resulted in amino acid changes at ACE2 protein were used for reconstruction of different receptor and molecular docking.

Dunbrak rotamer library was used to substitute targeted amino acids residues with those that are prevalent in Iranian population [23,24]. The most probable amino acid conformation, which is provided by UCSF Chimera software, was used for substituting desired amino acids. After mutagenesis, all atomic clashes were resolved using UCSF-Chimera’s energy minimization.

### Molecular docking & interaction analysis

The ClusPro web server (https://cluspro.org/login.php) [25] was used to dock the virus glycoprotein with ACE2 variants. The results of energy parameter set is reported as a total interaction energy (hydrophobic+hydrogenic+electrostatic). Models of docked macromolecules were ranked and reported by cluster size with lowest score.

The results of docking SARS-CoV-2 glycoprotein with the ACE2 variants were analyzed by MGLTools 1.5.6 software (The Scripps Research Institute, CA, USA) for determining ACE2 amino acids residues involved in interaction with the viral glycoprotein. The interaction analysis were done according to visual molecular dynamics (VDW) scaling factor 1 Å and the amino acid position(s) of all binding sites were investigated and validated in UniProt database regarding the molecular domains [26].

### Literature review of ACE2 polymorphism at different geographical regions

The scholar databases, including Google Scholar, PubMed and Scopus. The search terms were *polymorphism*, *Population*, *ACE2* or ‘*angiotensin I converting enzyme 2’*, *SNO* or ‘*Single nucleotide polymorphism’* (SNP). Furthermore, ClinVar [27] and single nucleotide polymorphism database (dbSNP) [28] databases were searched for known variations at ACE2. The results of literature search and further ACE2 variants was compared with the Iranian population exome sequencing at Iranome database.

## Results

### Structure & polymorphisms of ACE2 gene among Iranian

**Figure 1.**
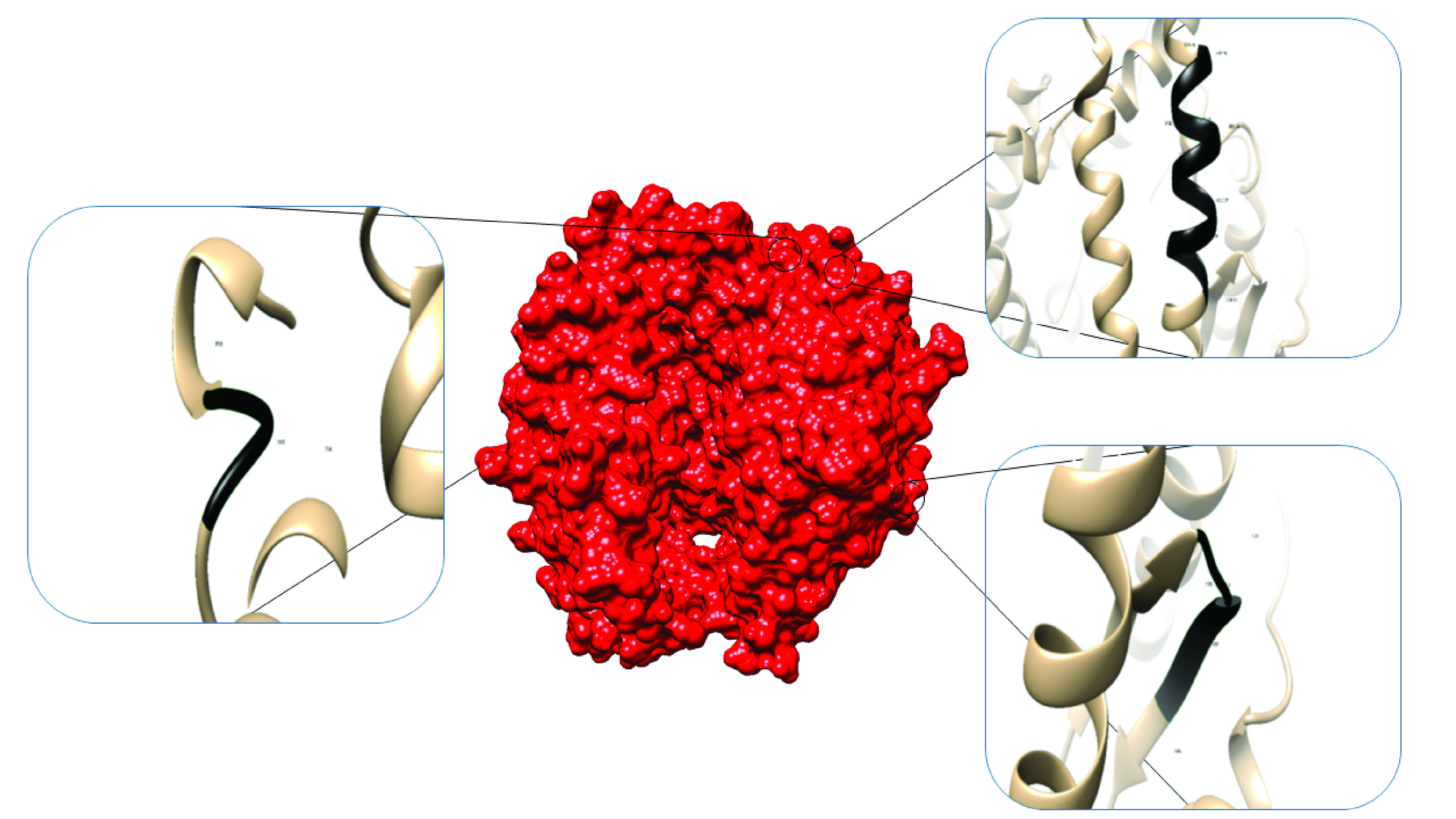
Three severe acute respiratory syndrome coronavirus binding sites resides within ACE2. Right top: amino acids 30–41 (DKFNHEAEDLFY), right bottom: amino acids 82–84 (MYP) and left center: amino acids 353–357 (KGDFR).

Native human ACE2 crystallographic structure with 615 amino acid length containing SARS binding domains were retrieved from PDB database. The protein comprised three regions responsible for interaction with SARS-CoV spike glycoprotein. These were amino acids 30–41, 82–84 and 353–357 (Figure 1). Therefore, it was though that same regions or neighboring amino acids would be involved in the interaction of ACE2 with SARS-CoV-2. In addition, only missense mutations with amino acid substitutions at ACE2 protein were chosen for further study.

Table 1 shows missense mutations, their annotation and frequency at ACE2 gene of Iranian genome database, Iranome. Mutations T15582298C at exon 17 responsible of N334D and mutation C15582333T at exon 17 responsible for R786W with 0.63 and 0.56% were frequent polymorphisms. Only substitutions within the ACE2 crystallographic structure were used for reconstruction of the mutant protein. Accordingly, substitutions at position beyond 661 were ignored.

**Table 1.** Annotation of missense mutations at ACE2 gene located at chromosome X of Iranian population.

Variant	Position	Protein consequence	Transcript consequence	Exon n	Allele count	Allele n	Number of homozygotes	n of heterozygotes	n of hemizygotes	Allele frequency (%)
15591578 C/G	15591578	Val485Leu	c.1453G >C	11	3	1600	0	3	-1	0.19
15607567 T/C (rs750145841)	15607567	Tyr199Cys	c.596A >G	5	2	1600	1	0	-1	0.13
15599413 G/A	15599413	Thr334Met	c.1001C >T	8	3	1598	1	1	-1	0.19
15599422 G/A	15599422	Ser331Phe	c.992C >T	8	1	1598	0	1	-1	0.06
15593877 A/C	15593877	Phe452Val	c.1354T >G	10	1	1600	0	1	-1	0.06
15580089 A/G	15580089	Ile786Thr	c.2357T >C	18	3	1598	1	1	-1	0.19
15618856 T/C (rs759162332)	15618856	Gln60Arg	c.179A >G	1	2	1600	1	0	-1	0.13
15591550 T/A (rs765152220)	15591550	Asp494Val	c.1481A >T	11	1	1600	0	1	-1	0.06
15607489 T/C	15607489	Asp225Gly	c.674A >G	5	2	1600	1	0	-1	0.13
15582298 T/C (rs41303171)	15582298	Asn720Asp	c.2158A >G	17	10	1600	2	6	-1	0.63
15582334 G/A (rs776995986)	15582334	Arg708Trp	c.2122C >T	17	9	1596	4	1	-1	0.56
15582333 C/T (rs769062069)	15582333	Arg708Gln	c.2123G >A	17	3	1598	1	1	-1	0.19

### Affinity & site of interaction of SARS-CoV-2 spike glycoprotein to human wild-type & mutant ACE2

**Figure 2.**
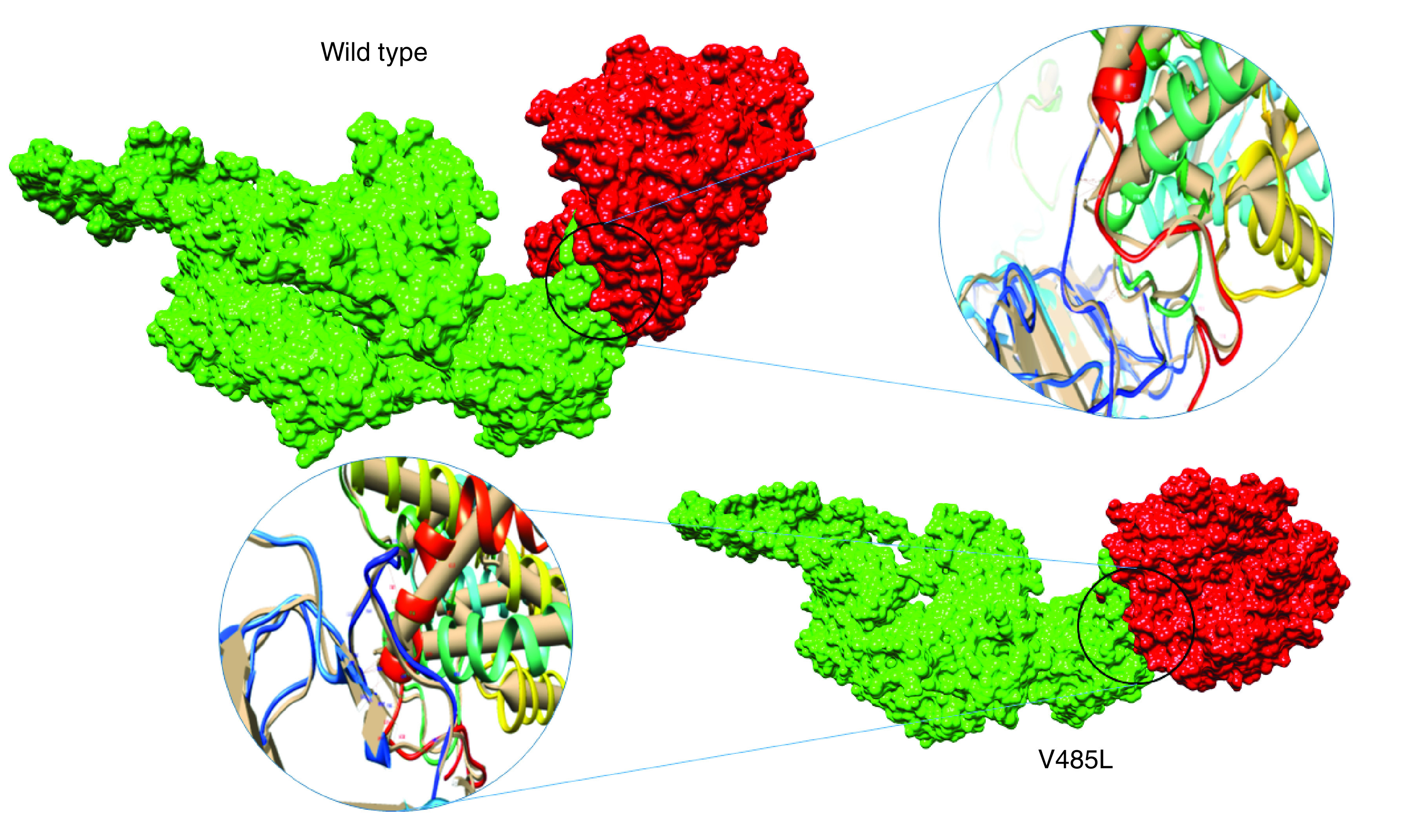
Schematic illustrations of interactions between severe acute respiratory syndrome coronavirus 2 and human ACE2 receptors. The residues involved in the interaction between wild-type receptor and viral ligand with <0.01 Å distance were Ser280, Leu156, Asp615 and Leu281 at ACE2. In addition, residues Asp597 and Gln598 were involved in the interaction between V485L mutant receptor and viral glycoprotein.

The interaction energy and affinity of SARS-CoV-2 to mentioned ACE2 variants are demonstrated in Table 2. As it is resulted, most of the ACE2 variants have same interaction energy with SARS-CoV-2 spike glycoprotein. However, the virus glycoprotein has a slightly higher affinity to ACE2 variant S331F. Interestingly, it was found that SARS-CoV-2 spike glycoprotein has much lower affinity to the human ACE2 polymorphism V485L (-972.2 Kcal.mol^-1^). It was also found that fewer close contacts were established between SARS-CoV-2 spike glycoprotein and ACE2 V485L mutant. Figure 2 shows individual residues involved in the interaction between the wild-type and V485L receptors and viral glycoprotein. The overlap residues within SARS-CoV-2 glycoprotein that are involved in the interaction with wild-type and V485L mutant of ACE2 are given in Supplementary Tables 1 & 2.

**Table 2.** Results of protein–protein docking and neighboring cluster members of severe acute respiratory syndrome coronavirus 2 spike glycoprotein docked with human ACE2 receptor.

Variant	Member	Representative (weighted score^†^)	
		Center^‡^ (Kcal.mol^-1^)	Lowest energy (Kcal.mol^-1^)
D225G	69	-1002.5	-1002.5
D494V	68	-1001.2	-1001.2
F452V	70	-1002.5	-1002.5
Q60R	68	-1002.5	-1002.5
S331F	74	-1000.9	-1003.7
T334M	68	-1002.5	-1002.5
V485L	56	-925.3	-972.2
Wild-type	70	-1002.5	-1002.5
Y199C	79	-1000.2	-1001.2

† *E* = 0.40*Erep* ± 0.4*Eatt* + 600*Elec* + 1.00*EDARS* (25).

‡ Greedy clustering of ligand position with a 9 Å C-alpha’s rmsd radius for finding ligand position with the most ‘neighbors’ in 9 Å.

att: Lennard-Jones repulsive energies; DARS: Decoy as reference state; Elec: Electrostatic; rep: Lennard-Jones attractive energies; rmsd: Root-mean-square deviation.

### ACE2 polymorphisms & their clinical importance

The known SNPs of ACE2 among some populations is given in Table 3. Most polymorphisms are located within introns regions (dbSNP data). In comparison with those provided in the present study, other populations had different polymorphisms at ACE2 coding sequence. Accordingly, such SNPs were involved in hypertension symptoms in patients.

**Table 3.** Different polymorphisms of ACE2 in literatures from different populations and single nucleotide polymorphism database.

Polymorphism of ACE2	Patient	Control	Population		Ref.
rs1514283 rs4646155 rs4646176 rs2285666 rs879922	1024 (male, 510; female, 514) EH patients	956 (male, 296; female, 660) NT controls	China, Han		[29]
rs2074192 rs2106809	647 patients (347 females and 300 males) with newly diagnosed mild-to-moderate EH	–	China–Chinese Han		[30]
rs4646127 rs2158082 rs5936011 rs6629110 rs4830983 rs5936029	–	–	EAS populations		[31]
rs4343; GG and GA genotypes	125 ACS patients with 77 men and 48 women kept on captopril 25 mg twice daily dosage	125 patients with 76 men and 49 women received no captopril (control study)	Iraq		[32]
rs2074192 rs233575 rs2158083 rs233575 rs2158083 rs233575 rs2074192 rs2158083	Participants in the NDIT cohort study (n = 555)	–	In males of European descent – In French Canadian males –In females of European descent		[33]
rs233566-rs233576 rs714205 rs757066 rs908004 rs963447 rs971249 rs971250 rs979848 rs1132186	–	–	–	dbSNP	

ACS: Acute coronary syndrome; dbSNP: Single nucleotide polymorphism database; EAS: East Asian; EH: Essential hypertension; NDIT: Nicotine dependence in teens; NT: Normotensive.

## Discussion

The SARS-CoV-2 is a novel virus that can cause severe respiratory disease in humans. The virus uses its glycoprotein to bind to the cell surface receptor, ACE2 [29]. ACE2 is a critical regulator of the renin–angiotensin system (RAS) that balances the amount of fluid in different organs such as the lungs [30]. ACE2 is a type I transmembrane glycoprotein and consists of 805 amino acids. ACE2 has two domains that include amino-terminal catalytic domain and the carboxyl-terminal domain. The catalytic domain of ACE2 is a zinc metallo-peptidase domain that actually constitutes its active site [15]. According to Cryo-EM study of the SARS-CoV-2 spike glycoprotein, it was shown that the virus had a higher affinity to ACE2 and binding to it is crucial for viral entrance [31]. The study on the role of genetic vaiants of host factors in SARS-CoV-2 infection continues [32,33]. It has been reported that certain genetic variations of ACE2 may affect the susceptibility to SARS-CoV-2 [34,35], the type of variants, their frequency in different populations [36] and their effect on the affinity of viral glycoprotein are not well established.

The receptor binding domain of SARS-CoV-2 spike glycoprotein, S1 (residues 318–510) has a higher ACE2 affinity than the complete S1 domain (residues 12–672) [37]. Nonetheless, residues 479 and 487 are essential to the successful link of the virus to ACE2. At ACE2, changes in lysine 31 and tyrosine 41, residues 82–84 and 353–357 affect its interaction with the S1 viral domain [38]. As a result, we examined the changes in ACE2 protein in the Iranian population genome to assess the affinity of viral spike glycoprotein to the host receptor. Two variants, T15582298C and C15582333 T, located at exon 17 with 0.63 and 0.56% of allele frequencies, were prevalent among the Iranian population. The literature review did not show any evidence of the same substitutions in other populations. Given that these SNPs have been identified in the Iranian population, they may be important in conferring natural resistance or have roles in the level of gene expression and function of ACE2. Although the genetic basis of ACE2 and its function in different populations are not clear, variations in ACE2 can affect the binding of the virus to ACE2 and the sensitivity of the host. Gomez *et al.* found no associations between ACE2 polymorphism and the disease outcome [33]. Nevertheless, the importance of ACE2 polymorphisms and their effects on SARS-CoV-2 spike glycoprotein affinity remained to be investigated in a certain population.

In a systematic study of 1700 variants of ACE2 from the China Metabolic Analytics Project under review and 1KGP (1000 Genome Project) databases, the authors suggested that there was no proof of a natural resistant ACE2 mutant in different populations [39]. In another analysis, it was also shown that rs73635825 (S19P) and rs143936283 (E329 G) variants affect the interaction of ACE2 with SARS-CoV-2 spike protein [40]. Natural ACE2 variants have also reported that are supposed to alter the susceptibility of the host. Stawiski *et al.* investigated variants, including S19P, I21V, E23 K, K26R, T27A, N64 K, T92I, Q102P and H378R, which increase susceptibility to virus infection. On the other hand, other ACE2 variants such as K31R, N33I, H34R, E35 K, E37 K, D38V, Y50F, N51S, M62V, K68E, F72V, Y83H, G326E, G352V, D355N, Q388L and D509Y reduced the binding affinity of SARS-CoV-2 spike glycoprotein to ACE2. It should be noted that these variants are rare in populations [17]. In addition, the genetic factors and age of SARS-CoV-2 hosts can also affect the susceptibility to infection [29,41]. On the other hand, studies have shown that there is no association between ACE2 variants and SARS-CoV virus susceptibility [42,43]. It is important to note that the expression of ACE2 may change under different circumstances, including cigarette smoking, diet and certain diseases such as pulmonary and cardiovascular diseases [30].

We examined the affinity and interaction site of SARS-CoV-2 spike glycoprotein with human wild-type and ACE2 mutants. According to our results, most ACE2 variants have the same interaction energy with SARS-CoV-2 spike glycoprotein. Nonetheless, the virus glycoprotein has a slightly higher affinity to ACE2 variant S331F (-1003.7 Kcal.mol^-1^). S331F may therefore increase the susceptibility of a population to SARS-CoV-2 infection. In addition, it was observed that viral glycoprotein had a far lower affinity to human ACE2 polymorphism V485L (-972.2 Kcal.mol^-1^). There was a less close interaction between SARS-CoV-2 spike glycoprotein and ACE2 V485L mutant in this regard. We propose that substitution of V485L for ACE2 may be a natural resistance mutation among the Iranian population. This may be due to conformational changes in the binding site within peptidase domain. All the studied variants of ACE2 were located at the N-terminal peptidase domain of ACE2. Our findings are presented based on other studies, which have been conducted by cryo–electron microscopy of the full-length human ACE2.

## Conclusion

ACE2 variants of the Iranian population had the same interaction energy with SARS-CoV-2 spike glycoprotein, most of which may not change the susceptibility to SARS-CoV-2 infection. In addition, S331F substitution can increase the affinity of viral glycoprotein to ACE2. This is due to the proximity of S331 to hotspot residues. This knowledge also suggests that the Iranian population with this particular variant is more susceptible to viral infection. Our data also suggest that the V485L variant of ACE2 may be a natural resistance mutation among the Iranian population. Considering the significance of these hotspots on ACE2, targeting these sites may be beneficial for treatment strategies.

**Summary points**

- The study presents an *in silico* analysis of affinity of severe acute respiratory syndrome coronavirus 2 spike glycoprotein to genetic variants of ACE2 in Iranian population.
- The virus glycoprotein had higher affinity to ACE2 variant S331F and people with same variants may experience severe viral infection.
- Severe acute respiratory syndrome coronavirus 2 spike glycoprotein has much lower affinity to the human ACE2 polymorphism V485L (-972.2 Kcal.mol-1).
- The V485L variant of ACE2 could be a natural resistance mutation among the Iranian population.
